# Variation and adaptation in the mechanical properties of primate molar tooth enamel

**DOI:** 10.1098/rsos.260677

**Published:** 2026-10-28

**Authors:** Frederick R. Foster, Adrian B. Mann, Paul J. Constantino, Robert S. Scott

**Affiliations:** ^1^ Department of Anthropology, The Ohio State University, Columbus, OH, USA; ^2^ Department of Material Science Engineering, Rutgers, New Brunswick, NJ, USA; ^3^ Department of Biomedical Engineering, Rutgers, New Brunswick, NJ, USA; ^4^ Department of Biology, Saint Michael's College, Colchester, VT, USA; ^5^ Department of Anthropology, Rutgers, New Brunswick, NJ, USA

**Keywords:** dental ecology, elastic modulus, hardness, nanoindentation, diet, primates, tooth enamel

## Abstract

Tooth enamel is a durable tissue with mechanical properties (stiffness and hardness) that vary throughout the teeth of primates with implications for dietary adaptation. Typically, estimations of tooth strength rely on averaged values of mechanical properties, which relegates variation within the crown that may be important for testing functional hypotheses. Here, we measured the mechanical properties of enamel in a taxonomically broad sample of primates and used generalized additive models to describe variation as gradients from the enamel-dentine juncture (EDJ) to the tooth surface. We found a sharp reduction in enamel stiffness at the EDJ and overall lower stiffness in the crown cervical margin across the sample. These regions are predicted to be vulnerable to fracture propagation during mastication, which reduced stiffness would help to mitigate. High hardness was identified at the cusp tips, consistent with increased wear resistance at the tooth surface. Large-bodied and hard-object feeding primates exemplified these patterns in our dataset. Our results suggest that mechanical properties in enamel vary to accommodate the maintenance of high strength throughout the cusp while simultaneously protecting vulnerable regions within from failure. This variation likely constitutes a key dietary adaptation with important implications for understanding dental function in medical and evolutionary contexts.

## 1. Introduction

Mastication is an early and vital step in the digestive process where nutrients and energy are unlocked from foods as they are broken down from their most mechanically resilient state. Tooth enamel is the primary tissue involved in food particle contact during mastication, undergoing millions of chew cycles and high bite forces while remaining structurally intact [1]. Poor mastication can occur when teeth are fractured, worn or infected, resulting in larger ingested food particles and either inefficient or incomplete absorption of nutrients and energy in the gut [2,3]. Additionally, if enamel fails via fracture or excessive wear occurs, dentine and the internal soft tissue of the tooth become open to infection and disease. One adaptive response to these problems lies in the mechanical properties of the enamel layer that evolved to be stiff enough to break down mechanically challenging foods while resisting failure owing to fracture and wear in the face of high bite forces and repetitive loading. Mechanical properties, including stiffness (i.e. the elastic modulus), hardness and toughness, are all relatively high in enamel in contrast to other materials, both biological and artificial, in which one of these properties is often enhanced at the expense of the others. The developmental and evolutionary mechanisms that facilitate this combination of mechanical properties remain unresolved and are a focus of current research in dental biomechanics.

Non-human primates are often used as a test group for hypotheses about dental biomechanics, because they are taxonomically diverse with dietary breadth that includes very hard and very tough foods, and because they provide insights into dietary adaptation in the human evolutionary past. Several studies have explored dietary adaptations in the teeth of human and non-human primates by mapping mechanical properties in the enamel layer [4–9]. A common finding of these studies is that stiffness and hardness vary throughout the enamel layer, but the significance of this variation is not well understood. One possibility is that variation in stiffness and hardness plays an adaptive role in tooth performance by increasing towards the enamel surface where particle contact occurs, leading to high stress, while decreasing towards the dentine layer where peak internal stress is observed [10–12]. However, the largest study of mechanical properties in primate tooth enamel to date found that stiffness and hardness had a weaker effect on tooth strength than the crown dimensions, suggesting that variation in mechanical properties is secondary to size and shape variables with respect to dietary adaptations for resisting tooth failure [5]. Constantino *et al.*'s [5] study focused on the central region of the enamel cusp tip, and an important next step is to assess variation in mechanical properties within other critical regions of the crown. These regions include the cervical margin of the crown and the enamel layer immediately adjacent to the enamel-dentine juncture (EDJ), where FEA models have identified locations of peak tensile stress under simulated bite forces [13–17]. Knowledge of variation in stiffness and hardness within these regions is critical to testing hypotheses about how the enamel layer functions in the context of dietary adaptation.

Enamel thickness has been linked to tooth crown strength in several theoretical [12,18–21] and empirical [22–26] studies. The proposed relationships can be expressed as functions of the mechanical properties of enamel and the dimensions of the crown (equations 1–3; figure 1). Recent studies have considered the role of relatively thick enamel in human and primate evolution, identifying it as a possible adaptation for crown strength through stress shielding, a mechanism that protects the internal, vulnerable parts of the tooth where fractures initiate and propagate [27,28]. A key aspect of stress shielding concerns the mechanical properties of the materials involved. In previous studies, average values for stiffness (the elastic modulus, E) and hardness (H) were used when solving equations 1–3, but this overlooks the fact that gradients in these properties are relevant to the effects of stress shielding [29,30]. Without an understanding of the variation in mechanical properties throughout the enamel layer, the role of enamel thickness and crown geometry in dietary adaptation cannot be fully understood.

**Figure 1.**
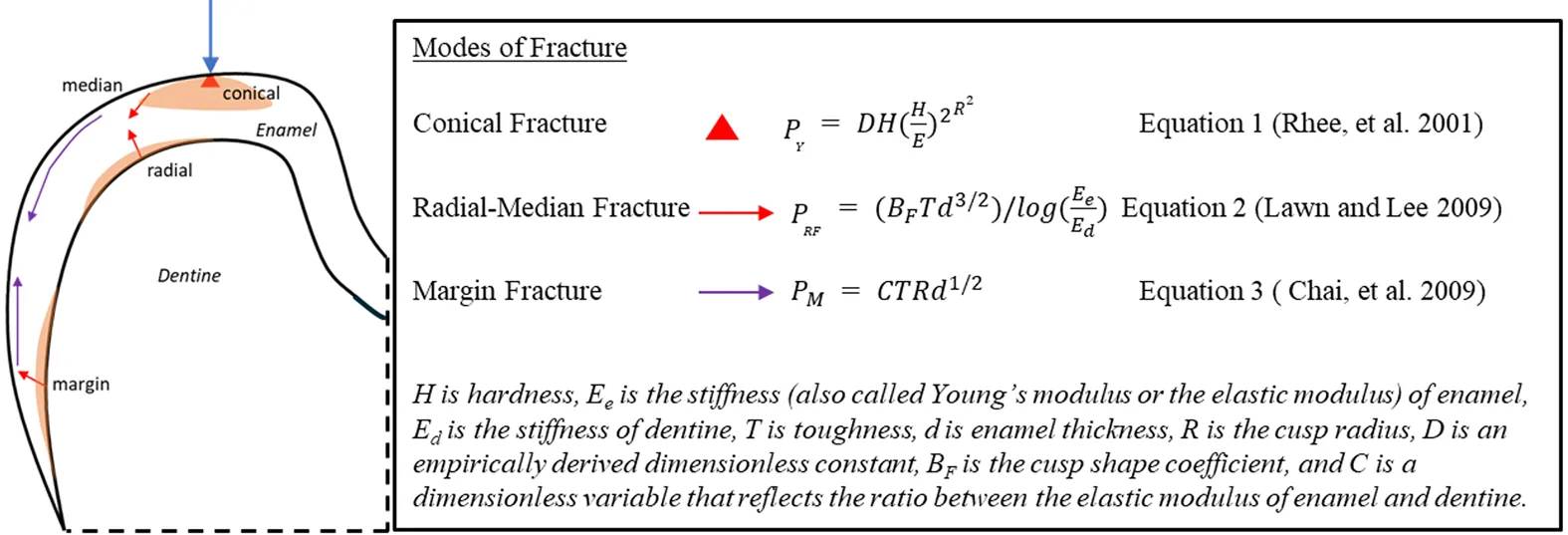
Schematic showing stress concentration and direction of fracture propagation within the cusp of a molar. The blue arrow indicates the direction of an occlusal load. Regions where stress is concentrated are shown in orange. The different modes of fracture are labelled, and their origins and directions of propagation are shown with arrows. The equations describing each mode of fracture are shown in the box. Purple arrows show the direction of propagation for fractures that reach the enamel surface. Tooth failure occurs when purple arrows intersect after moving entirely around the crown.

Here, we test two sets of hypotheses related to dietary adaptation in the mechanical properties of non-human primate tooth enamel. First, we test the hypothesis that significant interspecific differences exist in the average values for E and H of primate molar tooth enamel. We speculate that any significant variation in mechanical properties observed is related to diet and body size. Larger primates can generate higher bite forces, which increases the susceptibility of teeth to fracture and wear [31]. Similarly, primates with diets that include hard and/or tough foods can see increased rates of wear [32,33]. Given our small sample sizes, we do not explicitly test these predictions; however, we expect to see that larger primates and primates with more mechanically challenging diets will have higher average values of stiffness and hardness in their enamel compared with smaller bodied primates and primates with diets that do not regularly include hard or tough foods. Second, we test the hypothesis that variation in stiffness and hardness within the enamel layer is related to tooth function and failure. Specifically, we predict that regions of the crown that transmit bite forces and interact directly with food objects will be relatively harder and stiffer, while regions of the crown where peak internal tensile stress during chewing increases the likelihood of fracture initiation and propagation will be relatively less stiff. These regions include the wall and cervical margin of the crown and the EDJ beneath the cusp [12,29,30].

## 2. Material and methods

A sample of 22 lower molars with at least one representative from 10 different primate species was taken from scientific collections of deceased adult individuals (table 1). Whenever possible, second molars on the right side of the mandible were used. When lower second molars appeared damaged, excessively worn, or were absent, lower left second molars or lower first molars were used. All teeth used in this study were fixed in formalin before collection for use in this study. Four teeth were stored in the freezer at −80°C after drying at room temperature and fixing in formalin, before collection. Despite freezing and fixing in formalin, research suggests that the teeth used in this study are expected to retain structural and mechanical properties that are suitable for comparison across the sample [38,39].

**Table 1.** Description of specimens in the sample.

species	*n*	tooth	source	storage before collection	previous work
*Macaca mulatta*	7	LM_2_	A.J. Miller Lab, Dept. of Growth and Development, University of California	fixed in formalin	[34]
*Macaca* sp.	1	RM_2_	C.F. Ross Lab, Dept. of Organismal Biology, University of Chicago	fixed in formalin	—
*Colobus polykomos*	1	RM_2_			
*Lagothrix lagotricha*	2	RM_2_			
*Sapajus apella*	1	RM_1_			
*Papio anubis*	1	RM_2_	Southwest National Primate Research Center at the Texas Biomedical Research Institute	fixed in formalin (−40°C)	—
*Callithrix jacchus*	3	RM_2_			
*Pan troglodytes*	2	RM_2_			
*Colobus tephrosceles*	3	RM_2_	Kibale Hawk-Eagle and Ngogo	fixed in formalin	[35–37]
*Colobus guereza*	1	LM_2_	Chimpanzee Predation Collections		

### 2.1. Sample preparation

After collection, teeth were rinsed with acetone, stored in Hank's Balanced Salt Solution (HBSS) buffer [40] and removed approximately 48 hours before specimen preparation to dry at room temperature. Though drying is likely to affect the mechanical properties of enamel, the effect of drying is not expected to vary across specimens and thus not expected to affect comparative analyses [5,6]. After drying, each tooth was embedded in a two-part epoxy resin mould to stabilize the specimen during preparation and mechanical testing. Embedded teeth were then sectioned buccolingually through the distalmost cusps (see figure 2). This plane of section was chosen because it exposes the region of the enamel layer that is likely to experience the highest mechanical loading and resultant internal stress. Sectioning exposes, as close as possible, the cusp tips of the entoconid and hypoconid, which are in contact with food particles during crushing, grinding and shearing, and are located distally on the tooth where forces are higher during mastication. Sections were made buccolingually, as opposed to mesiodistally, because the mesial and distal ends of the tooth abut the first and third molars, respectively, which offer support during loading, whereas the mesial and buccal walls are not supported and thus likely to experience greater internal stress [41].

**Figure 2.**
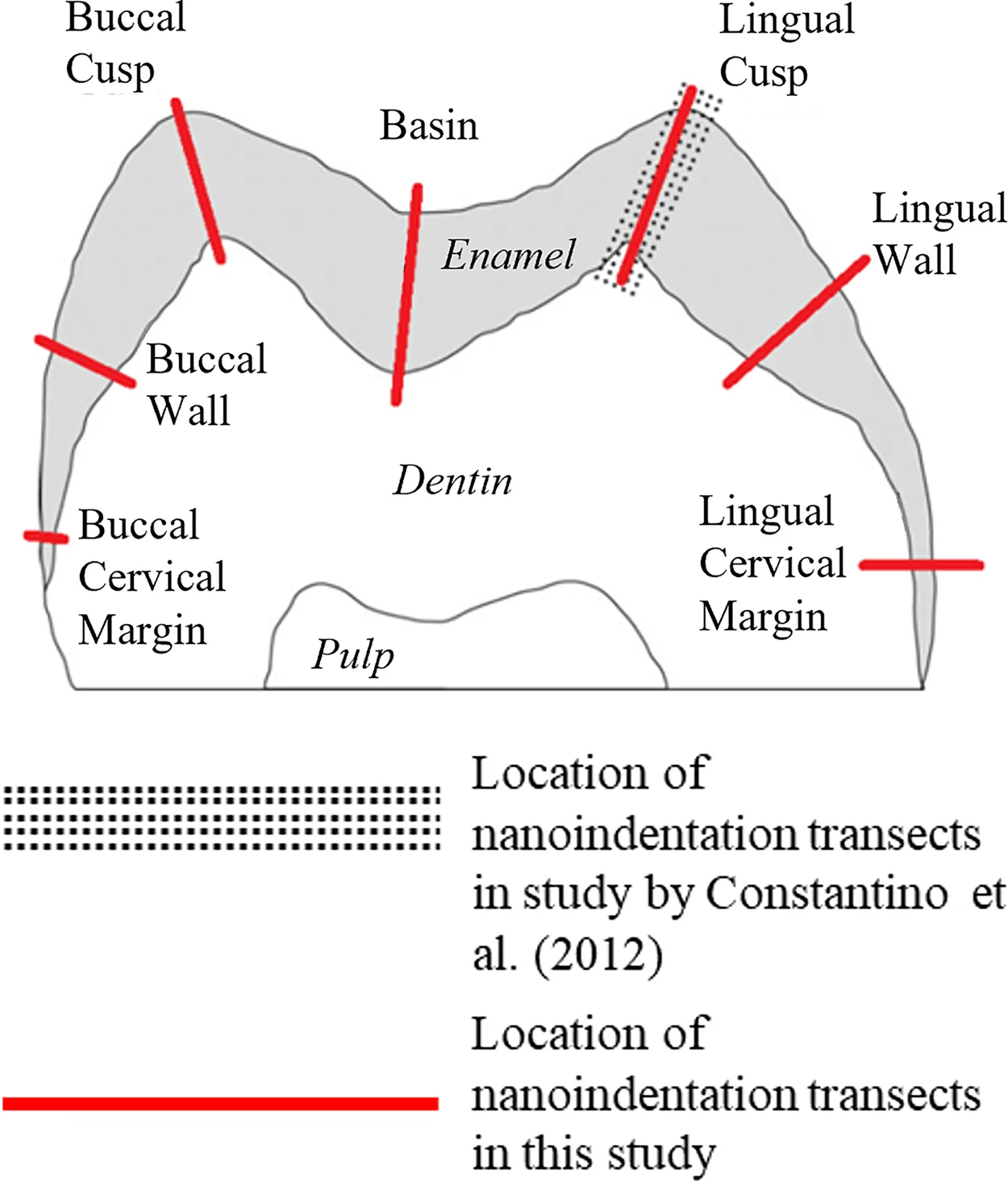
Nanoindentation transects through the enamel of a molar tooth in cross section (modified from [5]). Each transect has test locations spaced 20 µm apart, extending from the EDJ to the surface. Transects in this study run through regions that were not sampled in previous studies.

After sectioning, the exposed surface of each specimen was ground and polished using silicon carbide grinding paper and oil-based diamond slurries of diminishing grit sizes. Grinding pad grit sizes were 120, 400, 600, 800 and 1200 particles per unit area (FEPA standard). Diamond slurry particle sizes were 5, 2, 1 and 0.25 µm. Between polishing steps, each specimen was cleaned in an ultrasonic bath using DI water to remove debris. After polishing, another parallel section was made approximately 2 mm behind the prepared surface to create a section more suitable for mounting and testing. Each specimen was then mounted on a steel plate using a thin layer of epoxy. Before mounting, both the steel plate and the back of the thin section were ground using 1200 grit to create the appropriate surface roughness to maximize bond strength. Mounting the specimens to a steel plate ensures that flexion of the sample surface is eliminated, and that the measurement of mechanical properties is minimally affected by the compliance of the epoxy resin mould. The specimen and mounting plate were then wrapped in a piece of corrugated cardboard and placed in a vice with light pressure applied to ensure a strong bond between the specimen and the mounting plate.

### 2.2. Nanoindentation

Mechanical property testing was performed using a Hysitron Triboindenter^TM^ with a diamond Berkovich indenter tip. A displacement-limit indentation method was used, meaning each indent was constrained to a preset depth. A preset maximum depth of 400 nm was chosen, which is four orders of magnitude less than the sample thickness, ensuring that nanoindents only sample enamel and not the sample substrate. This is consistent with previous studies [5,8]. Each indent included three distinctly timed segments: a loading segment during which a load is applied to the specimen through the indenter tip (30 seconds), a hold segment during which the indenter tip is held at a constant depth of 400 nm (10 seconds), and an unloading segment during which the applied load is released as the indenter is retracted from the specimen (30 seconds). The applied force was changed by the nanoindenter to ensure that the depth of 400 nm was reached during the time allotted in the loading segment.

Indents were spaced 20 µm apart along linear transects running from the EDJ to the tooth surface at an angle approximately perpendicular to the EDJ. Each transect began approximately 60 µm in the dentine, so that the difference in mechanical properties across this boundary could be used to determine the beginning of each transect in the enamel. Choice of sampling location was constrained by features of the polished surface that were likely to affect the reliability of nanoindentation, including fractures, depressions, debris that was not removed during cleaning, and surface irregularities that were visible in the optical microscope.

### 2.3. Data analyses

First, variation across the regions of the crown was compared to identify general patterns in the distribution of mechanical properties within the molar enamel of primates. A parametric ANOVA and Tukey's HSD post hoc test were used to identify significant differences in the range of variation of E and H across the transects, when all specimens in the sample were pooled.

The changes in E and H with distance from the EDJ in enamel were evaluated using linear regression, generalized additive models (GAMs) and locally estimated scatterplot smoothing (LOESS) plots. GAMs and LOESS plots are useful for testing the hypotheses about changes in E and H with location within the enamel layer, because they are sensitive to local variation that may be functionally relevant. In this study, GAMs were used to fit nonlinear relationships between mechanical properties and distance through the enamel layer, to test the significance of these relationships, and to evaluate differences between species. Three different sets of GAMs were used to address the hypotheses of this study. First, GAMs were used to model the change in E and H with relative distance from the EDJ, with region of the crown and sample ID included as parametric terms to account for variation across these categories. LOESS plots were used to illustrate changes in E and H with relative distance through enamel within each region of the crown, for the entire sample.

Next, the relationship between mechanical properties and relative distance through enamel was modelled for the *M. mulatta* subsample, the largest for a single species in this study, to characterize the variation within a species. Four different models were considered when determining the best fit. First, a linear model was considered, with mechanical properties varying as a function of relative distance, and with region of the crown and specimen ID included as factors. Three GAMs were then considered, with each model building in complexity to determine which factors are driving the variation. The first GAM applied a smoothing function to relative distance with specimen ID as a factor. The next GAM added specimen ID as an interaction term to the smoothing function. This allows the model to independently fit smoothed relationships for each specimen, as opposed to a single smoothed relationship for all. The last GAM added the region of the crown as an additional factor.

The last set of GAMs focused on the hypotheses related to the load-bearing role of enamel during chewing. The data for each specimen were winnowed to include only the transect through the longest cusp preserved on the crown. Four different models were fitted to the data to evaluate the relationship between mechanical properties and relative distance from the EDJ, and to consider the factors that may be influencing this relationship. First, a linear regression model was fit with species included as a factor. Next, three GAMs were fit with each building in complexity. The first GAM applied a smoothing function to relative distance, with species included as a factor. The second GAM added an interaction effect owing to species to the smoothing function applied to relative distance, to address the possibility that gradients through the cusp vary by species. The third GAM added specimen ID (SID) as a factor.

## 3. Results

The elastic modulus and hardness were calculated using load-displacement curves and the Oliver & Pharr [42,43] method. Curves were examined for discontinuities and deviations from the expected parabolic shapes of the loading and unloading portions.

### 3.1. Mean elastic modulus and hardness

The mean values for E and H across all regions of the crown give an overall picture of the mechanical properties for each specimen in the sample and illustrate the range of variation within a species (table 2). The highest mean values of E were measured in one of the *Pan troglodytes* specimens (4X0315), followed by the *Sapajus apella* specimen, one of the *Lagothrix lagotricha* specimens (LLagF) and one of the *Macaca mulatta* specimens (MacacaG). The lowest mean values of E were measured in two of the *Callithrix jacchus* specimens (18495M and 28534M) and the *Papio anubis* specimen. The highest mean values for H were measured in one of the *C. jacchus* specimens (19577M), followed by the *S. apella* specimen. The lowest mean values for H were measured in one of the *P. troglodytes* specimens (4X0357), followed by one of the *M. mulatta* specimens (MacacaE) and the *P. anubis* specimen.

**Table 2.** Means and standard deviation for the elastic modulus and hardness of enamel in each specimen for measurements between 5% and 95% through the enamel layer.

sample	species	*n*	E (GPa)	H (GPa)
MacacaA	*Macaca mulatta*	96	91.0 ± 9.9	4.43 ± 0.65
MacacaB	*Macaca mulatta*	139	92.3 ± 9.6	4.52 ± 0.50
MacacaC	*Macaca mulatta*	211	99.8 ± 5.8	4.52 ± 0.39
MacacaE	*Macaca mulatta*	136	85.6 ± 10.3	4.02 ± 0.45
MacacaF	*Macaca mulatta*	181	84.6 ± 6.9	4.48 ± 0.44
MacacaG	*Macaca mulatta*	115	102.8 ± 7.2	4.53 ± 0.33
MacacaH	*Macaca mulatta*	136	90.0 ± 8.1	4.28 ± 0.37
MacacaSP	*Macaca* sp.	114	87.5 ± 7.4	4.34 ± 0.33
G83	*Papio anubis*	159	77.0 ± 10.8	4.12 ± 0.46
004-1	*Colobus guereza*	115	100.6 ± 7.7	4.48 ± 0.52
CPoly2	*Colobus polykomos*	143	93.8 ± 11.1	4.34 ± 0.45
PBadius111	*Colobus tephrosceles*	92	86.8 ± 9.4	4.44 ± 0.43
001-1	*Colobus tephrosceles*	118	99.1 ± 9.3	4.57 ± 0.43
007-1	*Colobus tephrosceles*	30	81.2 ± 6.7	4.38 ± 0.61
18495M	*Callithrix jacchus*	46	78.6 ± 6.1	4.18 ± 0.65
28534M	*Callithrix jacchus*	25	74.0 ± 6.5	4.34 ± 0.58
19577M	*Callithrix jacchus*	49	83.9 ± 4.8	4.95 ± 0.56
LLagM	*Lagothrix lagotricha*	72	93.6 ± 5.8	4.42 ± 0.42
LLagF	*Lagothrix lagotricha*	122	102.0 ± 6.9	4.56 ± 0.37
CApellaM1	*Sapajus apella*	84	103.0 ± 6.8	4.62 ± 0.53
4X0357	*Pan troglodytes*	74	87.0 ± 10.9	3.94 ± 0.53
4X0315	*Pan troglodytes*	126	104.6 ± 11.3	4.34 ± 0.50

In some specimens, the variation in E and H appears coupled. *Sapajus apella*, one of the *L. lagotricha*, and one of the *M. mulatta* (MacacaG) specimens have comparatively high mean values for both E and H, while the *P. anubis* specimen and one of the *P. troglodytes* specimens (4X0357) have lower mean E and H. Of the two *P. troglodytes* specimens, one has relatively low mean E and H (4X0357) while the other has comparatively high mean values (4X0135). In other cases, mean values of E and H diverge (*C. jacchus*, 19577M), with relatively low mean E along with high mean H.

### 3.2. Variation between regions of the crown

The results of the ANOVA test comparing variation in E and H across the regions of the crown are shown in figure 3. The crown cervical margin on the buccal and lingual sides had significantly lower values for E than all other regions of the crown but were not significantly different from each other. The buccal cusp had significantly higher average values of E compared with all other regions except the lingual wall. When H was compared by region, significant differences were found between all regions, except between the cusps on the buccal and lingual side, which were significantly harder than all other regions.

**Figure 3.**
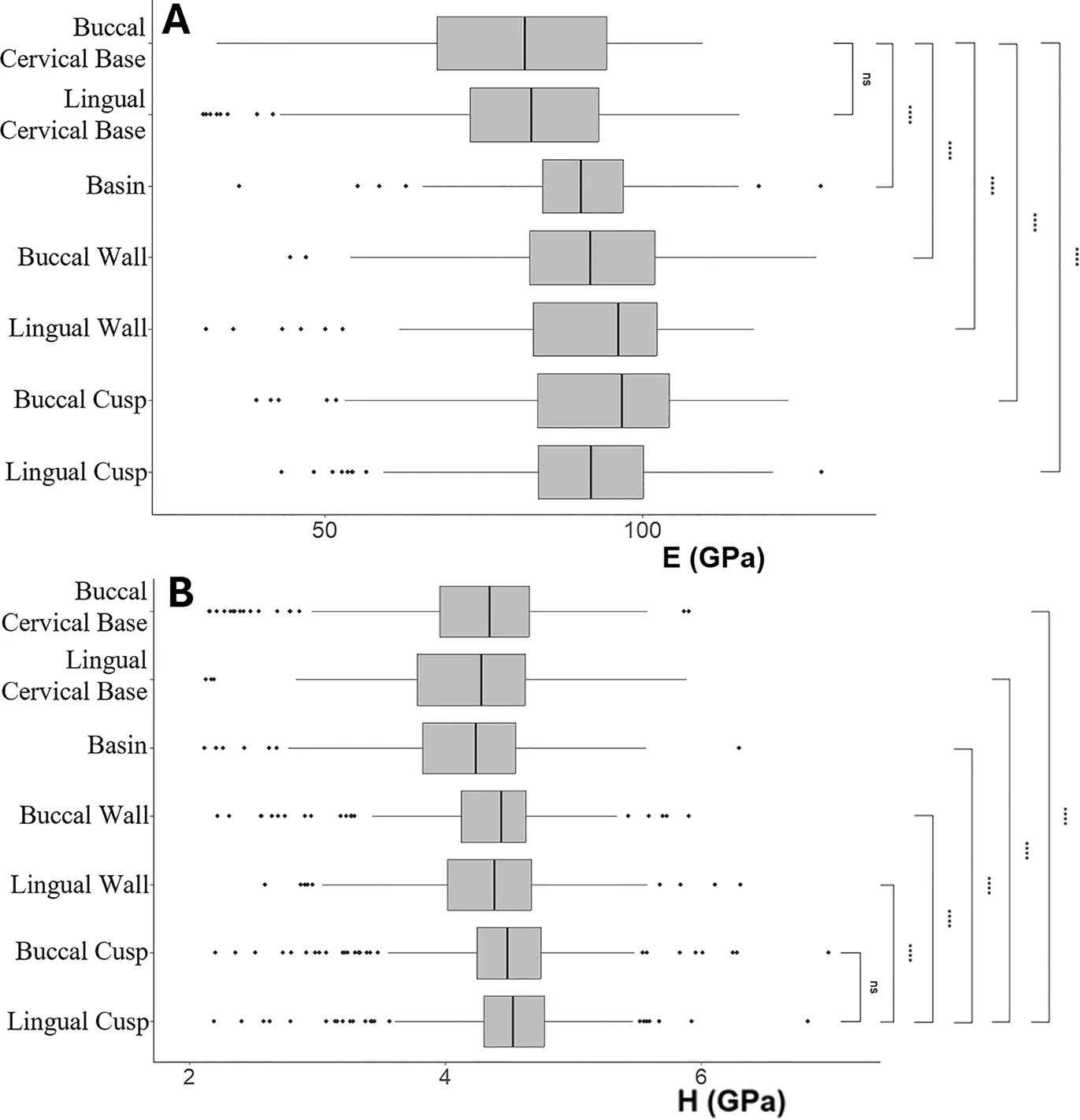
Range of variation in E (A) and H (B) of enamel in different regions of the crown. Significant differences are identified between the buccal base and all other regions (ns – not significant, **** *p* < 0.0001).

### 3.3. Mechanical property gradients in enamel

To test the hypotheses about fracture resistance and load-bearing capacity, we analysed the change in mechanical properties through the thickness of the enamel layer in different regions of the crown. Changes in mechanical properties are characterized here by plots of E and H with distance through the enamel layer for each specimen. Simple linear models for the relationships between E and H in each transect reveal increasing E and H through the enamel layer in all regions except the crown cervical margin on the buccal and lingual sides, where fewer data points result in relatively larger standard errors (figure 4). To account for deviations apparent in the linear models, GAMs were used to fit nonlinear relationships between mechanical properties and distance through the enamel layer, to test the significance of these relationships, and to evaluate differences between species.

**Figure 4.**
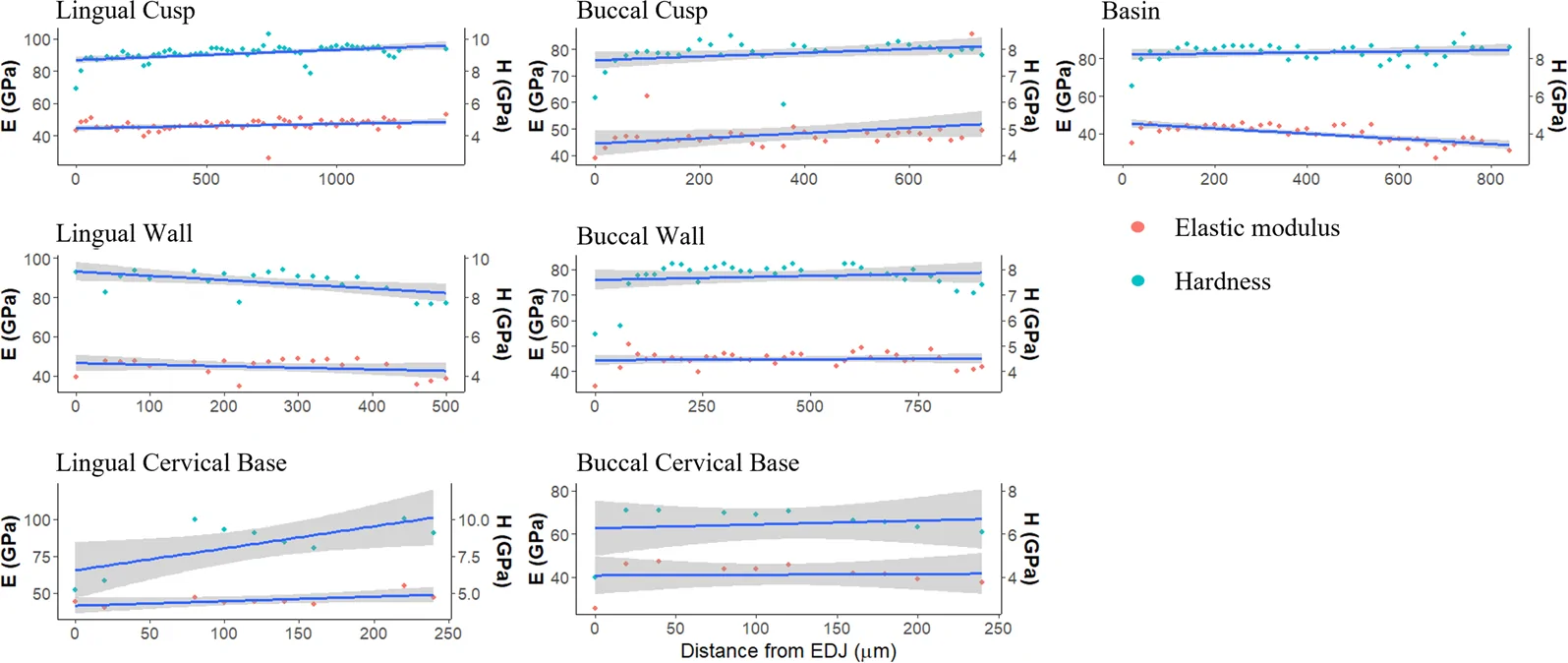
Elastic modulus and hardness versus distance through the enamel layer in all regions of the crown, for the MacacaF specimen (*M. mulatta*). The EDJ is at 0 µm, and the right-hand side is the enamel surface in each plot. The blue line shows a linear fit to the data, with the shaded region showing the standard error.

### 3.4. Gradients by region of the crown

The GAM used to evaluate the change in E and H with distance from the EDJ is given in table 3 where MP is either E or H depending on the property of interest, d is the relative distance through the enamel, R is the region of the crown and SID is the specimen ID. Also shown in table 3 is a simple, linear model with mechanical property as the response variable and relative distance as the predictor. Table 3 provides a metric comparison of the two models. The r-squared values give an indication of how well the model explains the data, and Akaike's Information Criteria (AIC) is used to select the model, where the lowest values indicate the model with the most explanatory power and least overall complexity.

**Table 3.** Model results for change in mechanical properties with distance through enamel.

	MP = E			MP = H		
model	*p*	r^2^	AIC	*p*	r^2^	AIC
linear: MP ∼ d + R + SID	<0.001	0.388	23 223.84	<0.001	0.199	4508.27
GAM: MP ∼ s(d) + R + SID	<0.001	0.455	22 878.54	<0.001	0.213	4458.43

Using a GAM offers an improvement over the linear fit, with a modest r-squared increase and AIC decrease for the relationships between both E and H and relative distance. The results of the GAM fit for E are shown in figure 5A for the smoothing function applied to relative distance. The smoothing function shows that, across all regions of the crown, a sharp increase in E occurs over the first 15% of the enamel layer after the EDJ. Thereafter, E may oscillate somewhat but cannot be readily distinguished from an overall slope of 0 for the rest of the thickness of the enamel layer.

**Figure 5.**
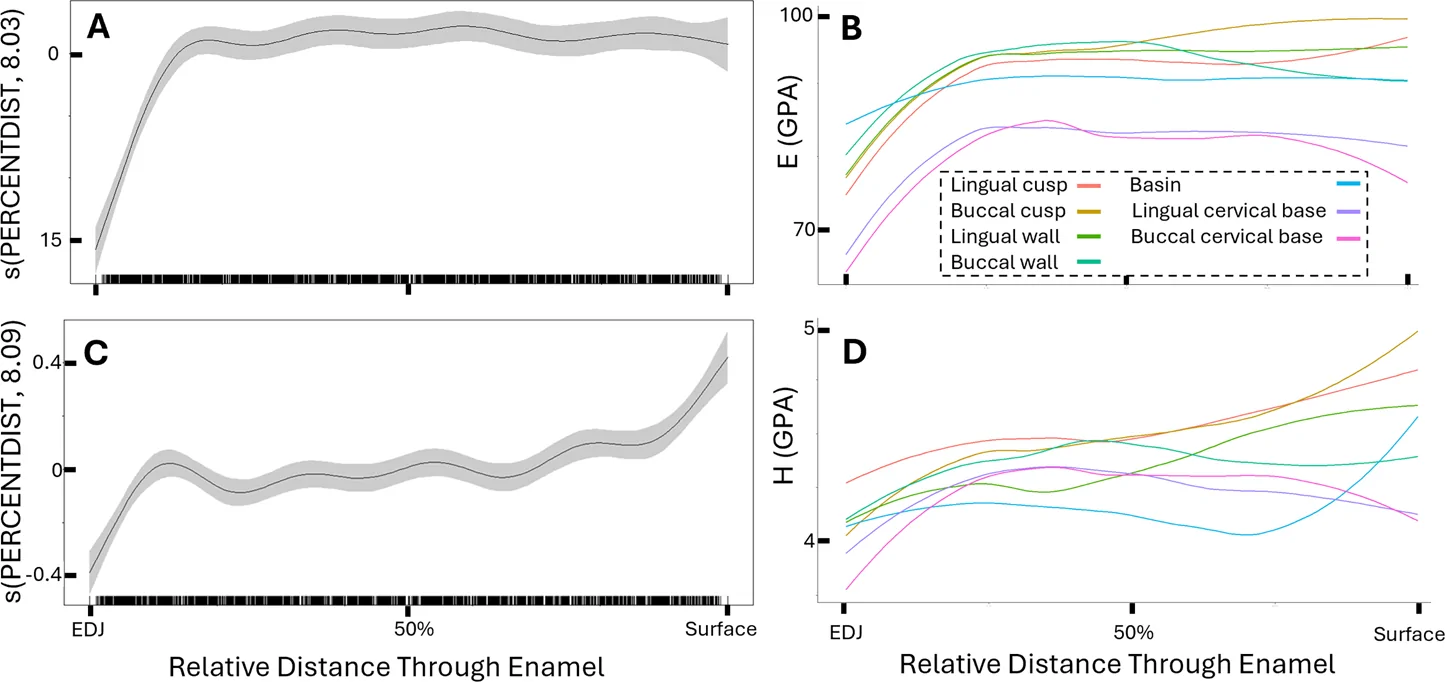
Nonlinear trends in E and H through enamel. Smoothing functions derived from the GAMs in table 4 for the relationship between E and relative distance (A), and H and relative distance (B), for all specimens and regions in the sample. The shape of the curve represents the best estimate of the change in E with distance through the enamel when all data in the sample are considered. The y-axis is in response units on the scale of the linear predictor, in this case PERCENTDIST, which is the relative distance from the EDJ. The y-axis label is the fitted function with the estimated degrees of freedom given in parentheses. LOESS plots for change in E (C) and H (D) with relative distance through the enamel for each region.

Figure 5A,B provides information for interpreting the LOESS plots for change in E with relative distance. The sharp increase in E just after the EDJ is evident in all regions of the crown except the basin, where the change is only weakly expressed. Both the lingual and buccal cervical margins have lower values for E overall than the other regions, which is consistent with the average values for each region described above. The buccal and lingual walls, and the buccal cusp have overall higher E than other regions, while the lingual cusp is intermediate.

The results of the GAM fit for H are shown in figure 5C for the smoothing function applied to relative distance. As with E, results of the GAM fit show a sharp increase in H in the first 15% of the enamel layer after the EDJ, before remaining approximately constant until the last 90% of the way through the thickness of enamel. Another sharp increase in H occurs over the last 10% of the enamel layer leading up to the surface, a change that does not occur clearly with E over the same distance. This shows that, while variation in E and H remain linked through the enamel layer, clear differences emerge when the relative depth within the enamel layer is considered more carefully.

Figure 5D shows LOESS plots for change in H with relative distance through enamel for each region of the crown. All regions exhibit an increase in H towards the enamel surface except the cervical margin of the crown on both the buccal and lingual sides, and the buccal wall, where H decreases moderately. Generally lower values of H are seen in the basin, though this region sees a steeper increase in H over the last 25% of the thickness when compared with all other regions except the buccal cusp, where it is comparable. The highest overall values in H are seen in the buccal and lingual cusps.

### 3.5. Gradients by species

The relationship between mechanical properties and relative distance through enamel was modelled for the *M. mulatta* subsample to characterize the behaviour within a comparatively well-sampled species. Four different models were considered when determining the best fit (table 4). First, a linear model was considered, with mechanical properties varying as a function of relative distance, with region of the crown and specimen ID included as factors. Three GAMs were considered as well, with each model building in complexity to determine which factors are driving the variation. The first GAM applied a smoothing function to relative distance with specimen ID as a factor. The next GAM added specimen ID as an interaction term to the smoothing function. This allows the model to independently fit smoothed relationships for each specimen, as opposed to a single smoothed relationship for all. The last GAM added the region of the crown as an additional factor.

**Table 4.** Model results for change in mechanical properties with relative distance through enamel for the *Macaca mulatta* subsample.

	MP = E		MP = H	
model	r^2^	AIC	r^2^	AIC
linear: MP ∼ d + R + SID	0.339	9552.12	0.173	1821.82
GAM1: MP ∼ s(d) + SID	0.428	9374.14	0.175	1821.98
GAM2: MP ∼ s(d, SID) + SID	0.406	9444.20	0.178	1826.73
GAM3: MP ∼ s(d, SID) + R + SID	0.465	9317.85	0.239	1733.91

AIC indicated GAM3 to be the best-fit model for both E and H. For the relationship between E and relative distance, the addition of an interaction effect owing to the smoothing function (GAM2) did not fit the data better than a model that did not include an interaction term (GAM1). When region of the crown is not included, specimen ID did not influence the curvilinear relationship between E and relative distance. Specimen ID was only important as an interaction term when region was included in the model (GAM3), suggesting that the curvilinear relationships between E and relative distance vary in specific regions across specimens in the subsample. For the relationship between H and relative distance, very little improvement was seen between the linear model and the GAMs that did not include region of the crown as a factor (GAM1 and GAM2). Significant improvement was only seen when region of the crown was included as a factor (GAM3), suggesting that variation across regions is principally responsible for the curvilinear nature of the relationship between H and relative distance through enamel.

For both E and H, the inclusion of ‘region of the crown’ as a factor has a significant impact on the fit of the model within a single species. This means that including all regions of the crown in comparisons across species in the sample may obscure meaningful differences. To account for this, and to focus attention on the hypotheses centred on the load-bearing role of enamel during chewing, the data for each specimen were winnowed to include only the transect through the longest cusp preserved on the crown. GAMs were again fitted to the data to evaluate differences in the curvilinear relationships between mechanical properties and relative distance through enamel. Four different models were fit to the data. The first was a linear relationship with Species (Sp) included as a factor. The next three GAMs were fit with each building in complexity. The first GAM applied a smoothing function to relative distance, with species included as a factor. The second GAM added an interaction effect owing to species to the smoothing function applied to relative distance. The third GAM added specimen ID (SID) as a factor. The results are summarized in table 5.

**Table 5.** Model results for change in mechanical properties with relative distance through enamel for the subsample that includes the longest transect through the cusp for each specimen.

	MP = E		MP = H	
model	r^2^	AIC	r^2^	AIC
linear: MP ∼ d + Sp	0.291	6131.60	0.106	1127.476
GAM1: MP ∼ s(d) + Sp	0.400	6001.88	0.118	1121.689
GAM2: MP ∼ s(d, Sp) + Sp	0.416	6006.43	0.187	1073.372
GAM3: MP ∼ s(d, Sp) + Sp + SID	0.848	4945.90	0.370	887.013

The best fit for the change in both E and H with relative distance was the GAM3 model that included an interaction effect owing to species in the smoothing function, and species and specimen ID as factors. The change in AIC and r-squared values when adding specimen ID as a factor shows that a large part of the variation in the sample is driven by differences in specimens. Despite this, species-level differences in the change in E and H with relative distance are indicated by the decrease in AIC when species is included as a factor.

Figure 6 shows the smoothing functions for change in E and H with relative distance by species used in the GAM3. All species except *Macaca* sp. and *P. anubis* show the same sharp increase in E over the first 15–20% of enamel thickness after the EDJ. The single specimens for *Macaca* sp. and *P. anubis* both have evidence of deterioration in their enamel near the EDJ, likely owing to tooth extraction from the mandible or sectioning, and data in this region were truncated. This may account for the absence of the increase in E after the EDJ in these specimens. All species have an uptick in E towards the enamel surface, except *Macaca* sp., *P. anubis* and *P. troglodytes*. The uptick is most pronounced in *M. mulatta*, *C. jacchus* and *C. tephrosceles*, while other species exhibit standard errors that obscure the change in E in this region. The greatest standard error is in *C. jacchus* (*n* = 3), and the smallest standard error is associated with *M. mulatta*, the largest sample (*n* = 7). In most species where there was a sharp increase in E after the EDJ, the slope of E through much of the enamel layer was 0. This is shown by the shaded standard error margins, which include 0 through most of the transect. Exceptions to this include *C. tephrosceles* and *C. polykomos*, where E increases gradually at about 60% through the thickness of enamel, and *P. troglodytes*, where E increases gradually until a slight decline at the enamel surface.

**Figure 6.**
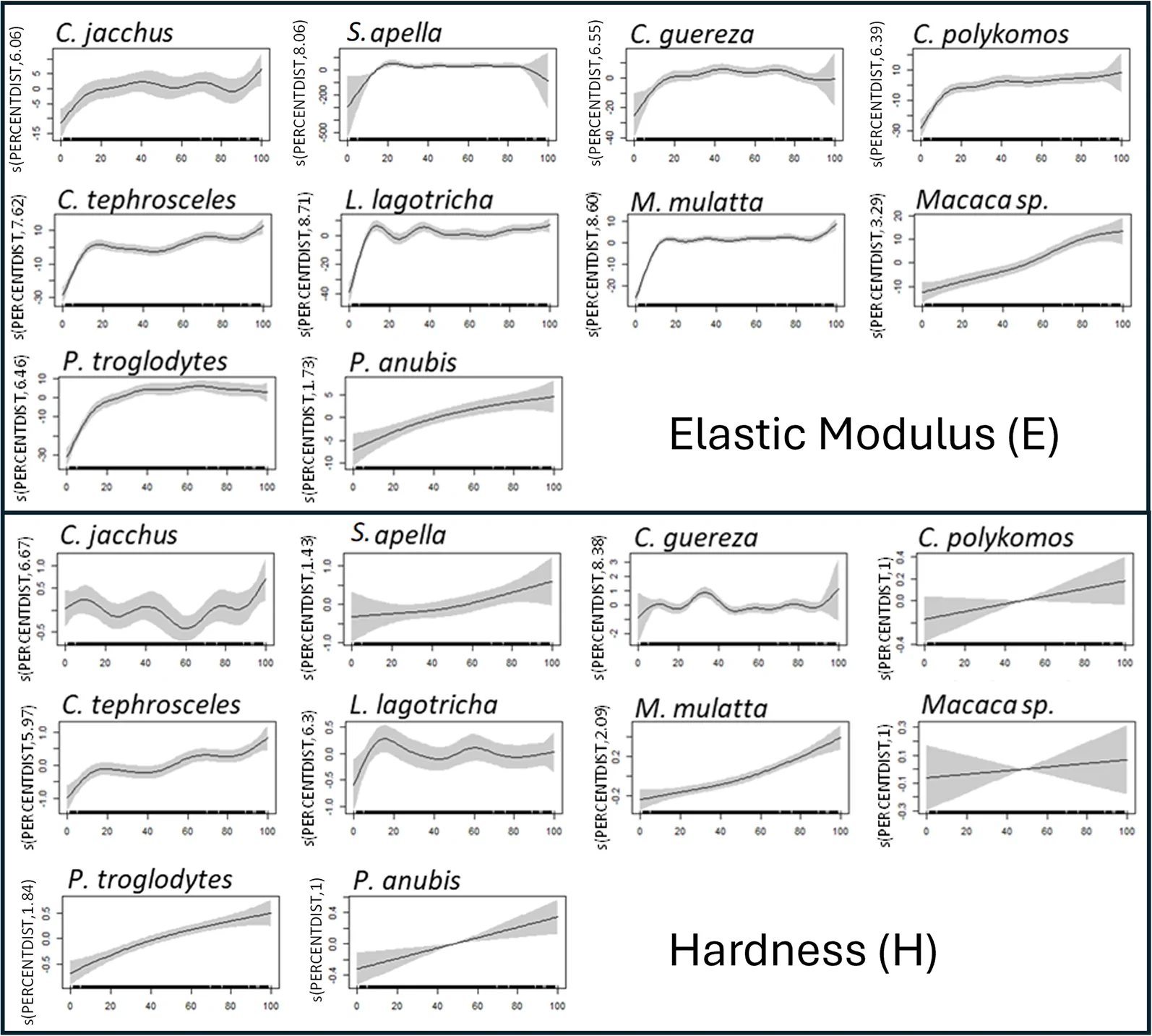
Smoothing functions for each species derived from the GAM3 in table 6 for change in E (top) and H (bottom) with relative distance through enamel (*x*-axis in each plot). Standard error is shown by the shaded lines. The *y*-axis labels show the fitted function with the estimated degrees of freedom in parenthesis shown for each species.

The results are different for the curvilinear relationship between H and relative distance (figure 6 (bottom)). The significant increase in H after the EDJ is only seen clearly in two species, *C. tephrosceles* and *L. lagotricha*. Three species, *Macaca* sp., *C. polykomos* and *P. anubis*, have changes in H through the enamel that appear to be linear, though wide error margins at the beginning and ends of the transects suggest that these regions may not have been sampled sufficiently to identify the change in this property. Wide standard errors are also seen for *C. jacchus* and *L. lagotricha*. The lowest standard error is seen in *C. guereza*, *C. tephrosceles*, *M. mulatta* and *P. troglodytes*. *Colobus guereza* has a highly curvilinear pattern for change in H with relative distance, but this is more likely an example of overfitting, where an outlier measurement at about 30% through the enamel layer has forced a more curvaceous fit than is otherwise warranted. *Macaca mulatta* has a clear increase in H with relative distance, with a tight standard error, and very little abrupt change throughout the transect. This may be the clearest signal of how H changes through the cusp and is similar to that seen in *P. troglodytes* and *C. tephrosceles*. Generally, H increases throughout the thickness of the cusp, but it appears to do so with more variation than is seen in E, requiring more individuals from a species to clearly resolve the relationship.

The LOESS plots for change in E and H with distance for each species are shown in figure 7 and are helpful for illustrating the relative values of mechanical properties at different points along the transect. *Pan troglodytes* has the highest values of E throughout nearly the entire thickness of enamel, while *C. jacchus* has the lowest. *Sapajus apella* also has comparatively high values of E throughout, followed by the three *Colobus* species and *L. lagotricha*. *Macaca mulatta* has median E values throughout, lying approximately halfway between *P. troglodytes* and *C. jacchus*. More variation across species is evident in the change in H with relative distance through the cusp. Unlike E, not all species exhibit low values of H near the EDJ, and this trend appears to be driven by *P. troglodytes* and three *Colobus* species in the cusp of the crown. Most species have an increase in H towards the enamel surface in the cusp, except *Macaca* sp., *C. polykomos*, *L. lagotricha* and *P. troglodytes*. The highest values at the enamel surface of the cusp are seen in *S. apella*, *C. tephrosceles* and *C. jacchus*.

**Figure 7.**
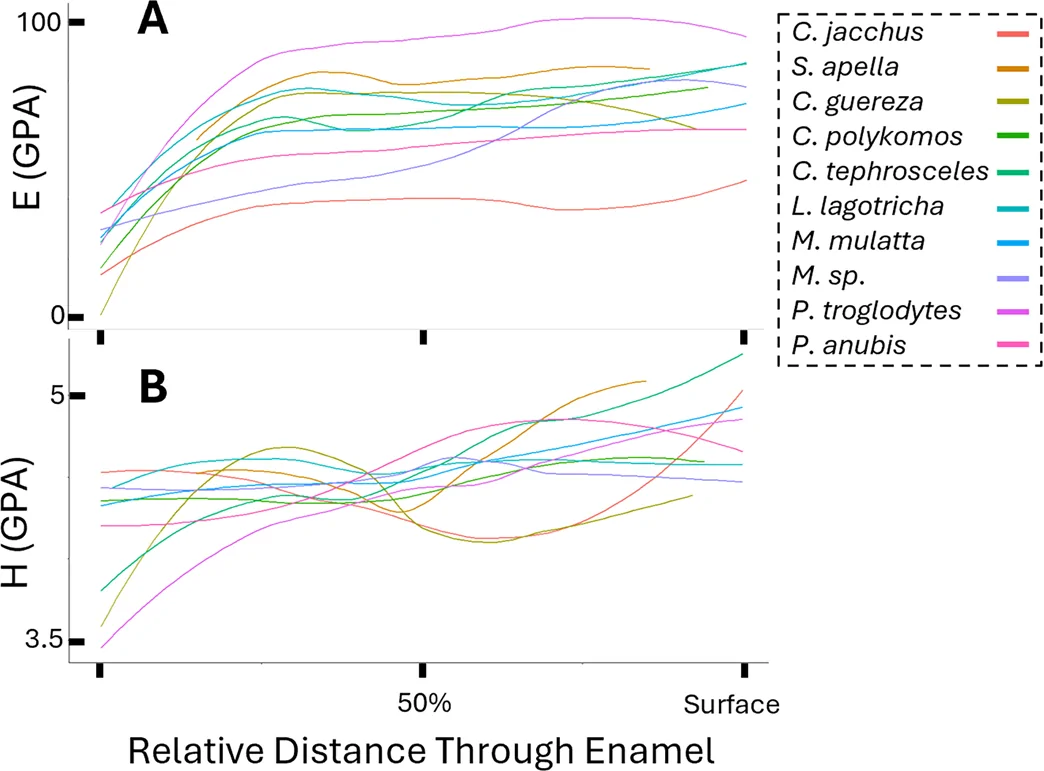
LOESS plots for change in E (A) and H (B) with relative distance through the enamel for each species.

## 4. Discussion

Tooth enamel needs to be hard and stiff to function effectively in mastication, but these properties create vulnerabilities elsewhere in the crown. Here, we show that hardness and stiffness vary within primate tooth enamel, consistent with our hypotheses related to tooth function, survivability and adaptation. Previous studies have identified variation in the mechanical properties of primate tooth enamel but argued that this variation is less important for dietary adaptation than cusp shape variables [5]. A recent investigation of catarrhines identified gradients in the hardness and stiffness of enamel through the crown and described these as functionally important [9]. By expanding the sampling strategy to include potentially sensitive regions of the crown, our analyses reveal signals of dietary adaptation in the mechanical properties of enamel that have not been fully appreciated despite being especially important for dental function and tooth survivability.

An important finding of this study is that the mechanical properties of molar enamel vary by region of the crown in primates. High E in the upper regions of the crown is consistent with the role of enamel in transmitting bite forces, while significantly lower E in the cervical margin of the crown may play an alternate and important role in dental function. FEM analyses of teeth with bite forces applied at an angle with respect to the root-crown axis have shown that peak internal stress occurs in the lower third of the enamel crown [13–16]. Primate jaw kinematics are complex, involving off-axis loading angles on teeth during chewing [44,45), causing repeated and regular occurrence of elevated internal stress. Equation 3 above shows this, where the force required to drive margin fractures through the base of the crown is given as a function of the numerical constant, C, which is an estimate of the hoop stress imposed by the more pliable dentine on relatively stiffer enamel owing to the elastic mismatch between the two materials [19]. Our finding that E is significantly lower in the cervical margin of the crown supports the hypothesis that adaptation has acted to reduce the elastic mismatch in regions of enamel that are vulnerable to peak internal stress and, in this case, the propagation of margin fractures.

Mean H in the buccal and lingual cusps was found to be significantly higher than in other regions of the crown, and this may play an adaptive role in wear resistance. Wear is the dominant mode of enamel failure for animals that eat tough foods, as well as small and hard foods, and for those foods that include other particles such as grit and potentially phytoliths in the diet [46–48]. High hardness is typically associated with wear resistance in materials, and this is especially true for small particles that indent and slide across the tooth surface [49,50]. Significantly higher H in the cusps may represent an adaptation for wear resistance in these regions, where wear occurs most frequently. Moreover, higher mean H in the cusps could indicate the maintenance of wear resistance in enamel at the occlusal surface, as wear accrues. Wear resistance would be mitigated if high H were present only at the enamel surface before declining as the surface layer was inevitably worn away. This is consistent with the results of Towle *et al.* [9], who found the highest values of H in the central regions of enamel cusps in a sample of primate molars that overlaps in some of the species from this study. Relatively higher H in the cusps compared with the other regions of the crown suggests that there may be maintenance of wear resistance within the tooth, either to slow the process of wear and extend the period during which the tooth functions most efficiently. Alternatively, variation in H throughout the crown may help to control wear rates and facilitate ‘sculpting’ of the occlusal surface into geometries that can enhance dental competence [51–53].

An overall picture of the change in E and H with relative distance through the enamel layer was found by fitting GAMs to the data from all specimens and transects in this study. This offers an advantage over previous studies that examined change in E and H by fitting splines [5,8], or by averaging the data over regions [4,7,9], because it captures changes that these methods may fail to identify. Our results found a marked increase in E over approximately 20% of the distance in enamel after the EDJ within the wall and cusps of the crown, consistent with some but not all previous studies in human and non-human primate tooth enamel. Studies that used spline fits to visualize the change in E through the enamel layer did not focus on changes near the EDJ [5,8]. This may be because they truncated the first 5% of the relative distance through enamel and used average values of E and H from the central portion of the enamel layer in their failure calculations. Towle *et al.* [9] failed to find a significant difference in modulus and hardness when comparing the inner, middle and outer regions of the crown in cross section, for a sample of catarrhines. By including the full range of transect data in our analyses and characterizing it using GAMs instead of averages, we were able to describe mechanical behaviour in critical regions and identify potentially meaningful signals of function and adaptation. Additionally, the lower values for E and higher values for H, highlighted in the GAMs, offer a possible explanation for the outliers seen in figure 3.

Previous work has pointed out that small sample sizes present a major confounding factor in efforts to compare mechanical properties of tooth enamel across species [4,5,7]. This study encounters similar issues for most species, but *M. mulatta* is represented by seven individuals, which is the largest sample size for a non-human primate in a study of this type, to date. As such, our dataset offers the most robust estimator of within-species variation in mechanical properties of tooth enamel. An ANOVA test (table 6) shows that the difference in the range of variation in E and H between the *M. mulatta* subsample and the rest of the sample is significant. This result is consistent with the hypothesis that differences in E and H between species exist, and suggests that these are worth considering as potential signals of dietary adaptation. However, figure 7 shows that there is still considerable overlap in E and H across species, supporting the previous suggestion that mechanical properties are highly conserved among primates [5,9].

**Table 6.** ANOVA results for significant differences in E and H between the variation in the *M. mulatta* subsample and variation in the other specimens.

	*p*	F_1,3097_
E	0.0067	7.372
H	0.0018	9.747

Within this study, high mean values for E and H by species were seen in *S. apella*, a primate with craniofacial features that can generate high bite forces [54] and a dental microwear pattern that is suggestive of at least occasional hard object feeding [55,56]. High E in the molar enamel of *S. apella* could facilitate the transmission of higher bite forces into relatively hard food items, with high H helping to resist wear and pitting. One of the *P. troglodytes* specimens (4X0315) had the highest overall values of E through the thickness of its cusps, and this species also had the greatest change in E over the first 25% of its enamel after the EDJ. High overall E with a large change at the EDJ is consistent with our adaptive hypothesis of variation in mechanical properties for an animal generating relatively high bite forces with respect to its body and tooth size [31]. High bite forces with broad cusps and thin enamel mean that the peak internal stress in enamel at the EDJ is likely to be large. In *P. troglodytes*, a sharp reduction in E in this region appears to be part of the adaptive package that helps to reduce stress in enamel and resist fracture.

Within the three *Colobus* species included in the sample, *C. guereza* had the highest mean values of E. *Colobus tephrosceles* had lower mean values of H than *C. guereza* and *C. polykomos,* which were similar. The digestive physiology of *Colobus* monkeys is adapted to process a fibrous diet, but this does not necessarily mean the foods that they eat are tough. *Colobus guereza* and *C. tephrosceles* are sympatric across part of their range with overlapping diets [57], but key differences may affect the mechanical demands placed on their teeth during chewing. The diets of *C. guereza* include significantly more tough, mature leaves than *C. tephrosceles,* who focus on the softer leaf petioles when ripe fruits are unavailable [58], allowing *C. guereza* to forage in regions that *C. tephrosceles* avoids [59]. This may explain why *C. tephrosceles* has the lowest mean E in the group, and why *C. guereza* has higher E throughout most of the thickness of its cusps. *Colobus tephrosceles* has the highest H at the enamel surface and the greatest increase in H through the cusps, when compared with the other colobus monkeys. It is possible that this is related to the high variability in the diet of *C. tephrosceles* compared with other colobus monkeys [60], making high H and wear resistance in the cusps adaptively beneficial when encountering harder seeds in ripe fruits.

The lowest mean values of E through all regions of the cusp were seen in *Callithrix jacchus*. This is not the case for H, where *C. jacchus* has relatively high mean H compared with the other species in the sample, with a sharp increase in H through the enamel in its cusp as it approaches the tooth surface. Low values for E may reflect the outcome of phylogenetic dwarfing in the callitrichids, resulting in the small body size and low bite forces [61,62], relaxing the selective pressure on E. High H, particularly at the enamel surface, may reflect the dietary ecology of *C. jacchus* as an active gummivore that extracts tree gum by gouging holes in tree trunks using its incisors [63]. This behaviour makes high H in enamel adaptively advantageous for wear resistance. However, this only explains the H observed in this study if the change in mechanical properties through the enamel is conserved across all tooth types, given that the observations in this study were generated from samples of *C. jacchus* molars. The conservation of mechanical properties across different tooth types in the same species has not been studied in detail and remains an important consideration when offering adaptive explanations for trends in the mechanical properties of enamel.

A key consideration when interpreting the results of indentation studies on teeth, including this study, is that enamel is an anisotropic material, meaning that its mechanical properties can vary depending on direction, orientation and spatial scale. Enamel has an internal microstructure comprised of prisms that extend along pathways of varying complexity from the EDJ to the tooth surface. Previous studies have implicated the prismatic microstructure as the cause of anisotropy in the mechanical properties of enamel [11,29,64,65]. This suggests that the plane of cross section used in our study could affect our results, as enamel prisms were more likely to be oriented roughly perpendicular to the direction of nanoindentation. Despite the small spatial scale of analysis involved in nanoindentation, each indent samples a volume of material that extends beyond the depth and breadth of the indent itself. This means that nanoindents will tend to sample a region that is influenced by more than one feature or orientation of the prismatic microstructure, which can mitigate its influence on measurements of mechanical properties. Nevertheless, microstructure remains challenging to control for and more work is warranted to explore its overall effects on nanoindentation studies. This potential confound, along with others, means that nanoindentation is not a simulation of the actual force and stress that enamel experiences during mastication. Nanoindents do not simulate the masticatory loading environment; rather, they provide a measurement of mechanical properties that can be used to describe how enamel performs under different loading conditions.

## 5. Conclusions

The mechanical properties of primate tooth enamel are an important component of their dietary ecology and are signals of adaptation. This includes not just the bulk or average values of E and H, but variation through the enamel crown as well. Here, we find evidence for adaptation within the primate molar crown based on how and where the E and H of enamel change. Primate molar enamel is significantly less stiff in the cervical margin of the crown, which facilitates greater deformation in this region as the expanding dentine increases the internal tensile stress in the enamel layer during chewing. The cusps remain comparatively stiff and hard, allowing for the transmission of bite forces into food particles while resisting failure owing to wear and fracture. A significant finding of this study is the sharp decrease in E in the enamel layer nearest to the EDJ, which is well characterized using GAMs to model the change in mechanical properties. This is likely an adaptation to minimize the elastic mismatch between enamel and dentine that places this vulnerable part of the anatomy under considerable duress during chewing, potentially driving fractures or even separating the two tissues. Low stiffness near the EDJ may be a critically important adaptation for primates, who are ecologically diverse and regular consumers of hard and tough foods, particularly during periods of resource stress when the maintenance of dental function is essential.

Some aspects of the variation in mechanical properties across primate species in the sample studied here are consistent with predictions from dietary ecology. Hard object feeding *S. apella* has the highest E, particularly in its cusps, even though it is intermediate in body size with respect to other primate species. Leaf-eating monkeys have generally higher E in their molar enamel with high H towards the cusp tips, which will aid in chewing tougher objects and prevent tooth failure over many chew cycles. The variation within the seven *M. mulatta* specimens in the sample suggests that species-level differences in mechanical properties may be detectable, and that signals of dietary adaptation are well worth considering. However, small sample sizes for individual species mean that adaptive interpretations should be tempered until more data are available, especially for rarer animals where hard tissue samples are coveted. For now, the results can be used to guide future studies that seek to identify signals of dietary adaptation in the dental ecology of these animals. Efforts to model the biomechanical behaviour of teeth in human and non-human primates, including fossil hominins, must include variation through the crown when assessing the adaptive role of enamel. The underlying biological, chemical and structural mechanisms that affect variation in E and H remain unknown and warrant further exploration.

## Data Availability

Data and relevant code for this research work are stored in GitHub: https://github.com/FFosterAnthro/FFoster-Enamel-Mech-Props and have been archived within the Zenodo repository [66].
